# Simultaneous Disruption of Mouse *ASIC1a, ASIC2* and *ASIC3* Genes Enhances Cutaneous Mechanosensitivity

**DOI:** 10.1371/journal.pone.0035225

**Published:** 2012-04-10

**Authors:** Sinyoung Kang, Jun Ho Jang, Margaret P. Price, Mamta Gautam, Christopher J. Benson, Huiyu Gong, Michael J. Welsh, Timothy J. Brennan

**Affiliations:** 1 Department of Anesthesia, Roy J. and Lucille A. Carver College of Medicine, University of Iowa, Iowa City, Iowa, United States of America; 2 Department of Internal Medicine, Roy J. and Lucille A. Carver College of Medicine, University of Iowa, Iowa City, Iowa, United States of America; 3 Department of Molecular Physiology and Biophysics, Roy J. and Lucille A. Carver College of Medicine, University of Iowa, Iowa City, Iowa, United States of America; 4 Department of Pharmacology, Roy J. and Lucille A. Carver College of Medicine, University of Iowa, Iowa City, Iowa, United States of America; 5 Howard Hughes Medical Institute, Howard Hughes Medical Institute, Chevy Chase, Maryland, United States of America; Duke University, United States of America

## Abstract

Three observations have suggested that acid-sensing ion channels (*ASIC*s) might be mammalian cutaneous mechanoreceptors; they are structurally related to *Caenorhabditis elegans* mechanoreceptors, they are localized in specialized cutaneous mechanosensory structures, and mechanical displacement generates an *ASIC*-dependent depolarization in some neurons. However, previous studies of mice bearing a single disrupted *ASIC* gene showed only subtle or no alterations in cutaneous mechanosensitivity. Because functional redundancy of *ASIC* subunits might explain limited phenotypic alterations, we hypothesized that disrupting multiple *ASIC* genes would markedly impair cutaneous mechanosensation. We found the opposite. In behavioral studies, mice with simultaneous disruptions of *ASIC1a*, -*2* and -*3* genes (triple-knockouts, TKOs) showed increased paw withdrawal frequencies when mechanically stimulated with von Frey filaments. Moreover, in single-fiber nerve recordings of cutaneous afferents, mechanical stimulation generated enhanced activity in A-mechanonociceptors of *ASIC* TKOs compared to wild-type mice. Responses of all other fiber types did not differ between the two genotypes. These data indicate that *ASIC* subunits influence cutaneous mechanosensitivity. However, it is unlikely that *ASIC*s directly transduce mechanical stimuli. We speculate that physical and/or functional association of *ASIC*s with other components of the mechanosensory transduction apparatus contributes to normal cutaneous mechanosensation.

## Introduction

Acid-sensing ion channels (*ASIC*s) represent an H^+^-gated subgroup of the degenerin/epithelial Na^+^ channel (DEG/ENaC) superfamily, which are Na^+^-selective or Na^+^-preferring cation channels sensitive to amiloride [1], [2]. Structural similarity between *ASIC*s and the degenerin mechanoreceptors (Mec-4, Mec-10 and Deg-1) from nematode *Caenorhabditis elegans* (*C. elegans*) [3]–[5] raised the possibility that *ASIC*s might also mediate mammalian mechanosensation. Almost all *ASIC* subunits are expressed in primary afferent sensory neurons [6], [7], and *ASIC2* and *ASIC3* immunoreactivities were detectable in specialized cutaneous mechanosensory structures such as Meissner corpuscles, Merkel cell neurite complexes and lanceolate nerve endings surrounding the hair shaft, supporting a possible role of *ASIC*s in cutaneous mechanosensation [8]–[10].

In previous studies, the involvement of *ASIC*s in cutaneous mechanosensation was evaluated by generating mice deficient in specific *ASIC* subunits and assessing the behavioral and electrophysiological phenotypes in these null mice [9]–[12]. However, in contrast to the striking impairment of touch sensitivity observed in *C. elegans* with null mutations in either Mec-4 or Mec-10 [4], [13], only subtle or no alterations in normal cutaneous mechanosensitivity were observed in mice with a single *ASIC* gene disruption. Ablation of genes for single *ASIC* subunits had no effect on the behavioral responses to mechanical stimuli [10], [11], [14]. Single fiber responses were affected in a variable manner by selective gene deletion. Reduced mechanosensitivity of the rapidly adapting (RAs) and slowly adapting low-threshold mechanoreceptors (SAs) was reported in *ASIC2* null mice [9]. In *ASIC3* null mice, RAs showed an enhanced mechanosensitivity, whereas Aδ-mechanonociceptors (Aδ-AMs) had a decreased sensitivity to mechanical sfƒtimuli [10]. No difference in the mechanosensitivity of afferents was found in *ASIC1* null mice [11]. In a study using cultured mouse dorsal root ganglia (DRG) neurons, no differences in amplitude or kinetics of mechanically activated currents were found between *ASIC2* or *ASIC3* single-null mice and wild-type (WT) mice [15].

*ASIC*s exist in heteromultimers in sensory neurons [7], and different *ASIC* subunits might function cooperatively in mechanosensation [16]. Considering the functional redundancy of *ASIC* subunits and the likelihood of functional compensation by remaining subunits that might occur during the development of *ASIC* single-knockout mice, analysis of animals missing multiple subunits should be of value in addressing the role of *ASIC*s in cutaneous mechanosensation. In this study, we therefore generated triple-knockout (TKO) mice with simultaneous disruptions of *ASIC1a*, -*2* and -*3* genes, and the cutaneous mechanosensitivity was evaluated in these animals using behavioral tests and single-fiber nerve recordings. Chemical responsiveness was evaluated using a combination of protons, lactate and ATP [17], and the effect of this chemical combination on the mechanical responses of cutaneous afferents was also evaluated in *ASIC* TKO and WT mice.

## Materials and Methods

### Generation of ASIC 1a/2/3 Null Mice

The generation of *ASIC1a*, -*2*, and -*3* null mice has been described [9], [10], [18]. Each of these lines was backcrossed for ten generations onto a C57BL/6J background to generate congenic lines of each. Once obtained, these congenic lines were crossed to one another to generate a congenic C57BL/6J line with the simultaneous disruption of *ASIC1a*, -*2*, and -*3* (*ASIC1a/2/3* null mice).

### Animal Care and Use

All experimental procedures were approved by The University of Iowa Animal Care and Use Committee, Iowa City, Iowa. The animals were treated in accordance with the Ethical Guidelines for Investigations of Experimental Pain in Conscious Animals issued by the International Association for the Study of Pain [19]. Combined behavioral and electrophysiological experiments were performed on 53 *ASIC* TKO mice (27 females and 26 males) and 59 age-matched WT C57BL/6J control mice (30 females and 29 males, Jackson Laboratory, Bar Harbor, ME). The weight of the mice was 20–30 gm, and the age was 6–12 weeks. One to five animals were housed in plastic cages in a climate-controlled room under a 12-h light/dark cycle.

### Electrophysiological Recordings in Isolated DRG Neurons

Lumbar (L4–6) DRG neurons from WT or *ASIC* TKO mice were collected and dissociated as described previously [7]. Briefly DRGs were treated with papain followed by collagenase/dispase, then gently triturated to isolated neurons, and then plated on poly-d-lysine and laminin-coated dishes. Culture medium contained F12 supplemented with heat inactivated serum (10%), penicillium-streptomycin and NGF (50ng/ml). Whole cell patch clamp recordings of DRG neurons were carried out at -70mV 18–24 h after isolation. Currents were filtered at 1 kHz and sampled at 2 kHz using the Axopatch 200B amplifier, Digidata 1200 and Clampex 8.2 (Axon Instruments, Union City, CA). Micropipettes (3–5 MΩ) were filled with internal solution (mM): 100 KCl, 10 EGTA, 40 Hepes, 5 MgCl_2_, pH 7.4 with KOH. External solutions contained (mM) 120 NaCl, 5 KCl, 1 MgCl_2_, 2 CaCl_2_, 10 HEPES, and 10 MES; pH was adjusted with tetramethylammonium hydroxide, and osmolarity was adjusted with tetramethylammonium chloride.

### Behavioral Study

Mice were acclimated to the testing environment for at least 5 days before any testing was performed. Withdrawal responses to mechanical and heat stimuli were tested in two separate groups of animals. Testing was performed at approximately the same time of day for each subject. The person performing the behavioral experiments was blinded to the genotype of animals.

To assess mechanosensitivity, mice were placed on a stainless steel mesh floor covered with a clear plastic cage top and allowed to acclimate. Calibrated von Frey filaments were applied to the plantar surface of the left hind paw near the medial heel, from least to greatest forces (0.6, 1.5, 3.4, 5.7, 13.7 and 26.8 mN). Each filament was applied five times at each time point, with 30 s between applications. Data were expressed as percent of paw withdrawal for each filament.

To assess heat withdrawal response, mice were placed on a heat-tempered glass floor (3 mm thick) covered with clear plastic cage top and allowed to acclimate. Withdrawal latencies to heat were assessed by applying a focused radiant heat source underneath a glass floor onto the proximal half of the plantar surface of the left hind paw. The heat stimulus was a light from a 50-W projector lamp, with an aperture diameter of 6 mm. Paw withdrawal latencies were measured to the nearest of 0.1 s. The latency time to evoke a withdrawal was determined with a cutoff value of 30 s. Before data collection, the intensity of the heat was adjusted to produce withdrawal latencies in WT mice of 23–25 s. Three trials 10 min apart were used to obtain the average paw withdrawal latency.

### Single-fiber Electrophysiology in the Mouse Skin-Nerve Preparation

Single-fiber recordings using the isolated skin-nerve preparation were performed in 16 *ASIC* TKO and 16 WT mice. The experimenters performing the recordings were blinded to the genotype.

The mouse saphenous skin-nerve *in vitro* preparation [20] was used to evaluate the receptive characteristics of cutaneous primary afferent fibers. Mice were euthanized in a carbon dioxide chamber, and the hair on the leg was clipped. The saphenous nerve and its innervated territory on the hairy skin of the hind paw were dissected free from muscles or tendons. To ensure a sufficient length of axons for recording, the nerve was dissected up to the lumbar plexus. The skin was then placed epidermal side down in the *in vitro* perfusion chamber, and was continuously superfused with modified Krebs-Hensleit (K-H) solution (in mM: 110.9 NaCl, 4.8 KCl, 2.5 CaCl_2_, 1.2 MgSO_4_, 1.2 KH_2_SO_4_, 24.4 NaHCO_3_ and 20.0 glucose, pH 7.4), which was saturated with a mixture of 95% oxygen and 5% carbon dioxide. The temperature of the bath solution was maintained at 32 ± 0.5°C. The nerve attached to the skin was then drawn through a small hole into the recording chamber containing a superficial layer of mineral oil and a bottom layer of modified K-H solution. The nerve was desheathed on a mirror stage, and small filaments were repeatedly split with sharpened forceps to allow single fiber recording to be made using extracellular gold-wire recording electrodes. Neural activity was amplified (DAM50, Harvard Apparatus, Holliston, MA), filtered, and displayed using standard techniques. Amplified signals were led to a digital oscilloscope and an audiomonitor and fed into PC computer via a data acquisition system (spike2/CED1401 program, Cambridge Electronic Design Ltd., Cambridge, UK). Action potentials collected on a computer were analyzed off-line with a template-matching function of Spike 2 software (Cambridge Electronic Design Ltd.). If more than one fiber was present in a recording, data were analyzed only if the unit’s action potential shape and amplitude could be easily discriminated from the other unit’s action potential.

The mechanoreceptive fields of afferent units were identified by probing the dermis side of the skin with a blunt glass rod; thus, mechanosensitive afferents were recorded. Only units with a clearly distinguished signal to noise ratio (greater than 2∶1) were further studied. Once the receptive field was identified, a 1 mN von Frey filament was applied perpendicular to the receptive field. Units responding to a 1 mN von Frey filament were classified as low-threshold mechanoreceptors. Those not responding to a 1 mN von Frey filament were classified as mechanonociceptors.

The experimental protocol was as follows: Once a single afferent was identified, the receptive field was isolated with a metal ring (5-mm internal diameter), which could seal by its own weight, was used. In some cases, inert silicone grease was added to ensure a waterproof seal. After a 2-min baseline recording, the mechanical response properties were measured using a servo force-controlled mechanical stimulator. Then, the modified K-H solution inside the ring was replaced with either test solution (15 mM lactic acid plus 5 µM ATP; pH 5.0; 32°C) or control solution (modified K-H solution; pH 7.4; 32°C) without any movement of the mechanical probe or ring. After 2-min application of the chemical solution, the same series of mechanical stimuli was applied to the receptive field in the presence of the applied solution. The conduction velocity (CV) was measured at the end of the experiment. The methods for evaluation of mechanosensitivity and CV are described in more detail below. To avoid sensitization/desensitization of nociceptors, fibers having receptive fields in the previously studied area were avoided for subsequent recording. The person recording the afferents was aware of the content of the solution, pH 5.0 lactic acid/ATP or control K-H solution.

To determine quantitative mechanosensitivity, a feedback-controlled constant-force mechanical stimulator (Series 300B Dual Mode Servo System, Aurora Scientific, Aurora, Ontario, Canada) [21] was used. A flat-ended cylindrical metal probe (tip diameter of 0.7 mm) attached to the tip of the stimulator arm was placed just close to the most sensitive spot of the receptive field so that no force was generated. After a 2-min baseline recording, a computer-controlled ascending series of square force stimuli (100-ms rise time, 1.9-s duration of sustained force plateau) was applied at 60-s intervals. Force stimuli of 5–40 mN range were used for low-threshold mechanoreceptors and 5–80 mN for mechanonociceptors.

To make the chemical test solution, NaHCO_3_ (24.4 mM), normally contained in modified K-H solution, was replaced with L-lactic acid (Sigma, St. Louis, MO; 85% to a final concentration of 15 mM), and the pH of lactic acid was adjusted to pH 5.0 with a few drops of 1N NaOH. The final sodium concentration was approximately 125 mM. Then, a frozen aliquot of the ATP (disodium salt, Sigma, St. Louis, MO) stock solution (1mM in saline, pH 4.0) was added to a final concentration of 5 µM. Lactic acid maintained its pH (pH 5.0) when mixed with the ATP stock solution. Aliquots of the ATP stock solution were stored at −80°C until use. The test solution was equilibrated with room air prior to application to the receptive field.

The CV of each unit was determined by electrical stimulation of the receptive field with bipolar platinum electrodes (0.3 mm in diameter, Surepure Chemetals Inc., Florham Park, NJ). Then the distance between the receptive field and the recording electrode (conduction distance) was divided by the latency of the action potential. Afferent fibers conducting slower than 1.2 m/s were classified as C-fibers; those conducting between 1.2 and 8.0 m/s as Aδ-fibers, and those conducting faster than 8 m/s as Aβ-fibers.

Fibers were further classified according to their mechanical threshold (low-threshold mechanoreceptors versus nociceptors, see above) and adaptation responses to sustained suprathreshold mechanical stimuli (SA fibers versus RA fibers) (Table 1). Aδ- and Aβ-fibers with a threshold of > 1 mN and a slowly adapting response to sustained suprathreshold force were considered as A-mechanonociceptors (AMs). Aδ-fibers with a threshold of ≤ 1 mN and a rapidly adapting response at the onset and offset of the force stimulus were classed as D-hair receptors (DHs). Aβ, low-threshold mechanoreceptors were further categorized as SAs if they responded throughout a sustained suprathreshold force stimulus or as RAs if they responded only at the onset and offset of the force stimulus.

**Table 1:** Fiber classification of mouse cutaneous mechanosensitive afferents.

	Conduction velocity	Adaptation characteristics	Mechanical threshold
Mechanosensitive C-nociceptor	< 1.2 m/s		
A-mechanonociceptor (AM)	≥ 1.2 m/s	Slow adaption	> 1 mN
D-hair receptor (DH)	1.2 – 8 m/s	Rapid adaption	≤ 1 mN
Rapidly adapting low-threshold mechanoreceptor (RA)	> 8 m/s	Rapid adaption	≤ 1 mN
Slowly adapting low-threshold mechanoreceptor (SA)	> 8 m/s	Slow adaption	≤ 1 mN

Action potentials collected on a computer were analyzed off-line with a template-matching function of Spike 2 software. If a unit discharged at a rate of 0.1 imp/s or more without any intentional stimuli, it was categorized as spontaneously active. Background activity of spontaneously active units was subtracted from any evoked response, thus assuming background activity was sustained during the stimulus period. For mechanical responses, the quantitative analysis of unit discharges was carried out by counting total action potentials in a response and by averaging responses in 0.3-s bins. For chemical responses, total action potentials were averaged during baseline and during chemical application. A unit was considered activated (responsive) when it discharged greater than 0.1 imp/s during chemical stimulation. If background activity was present, the unit was regarded as responsive if the activity was increased at least two standard deviations greater than the background activity during the chemical stimulation period.

### Statistical Analyses

Among behavioral data, paw withdrawal frequencies to von Frey filament were analyzed using two-way ANOVA with repeated measures on one factor; significant main effects of genotype or interactions were followed by a separate unpaired t-test at each force level. Differences between strains for single variables were compared using the unpaired t-test. Proportions between strains were compared using the χ^2^ test. The stimulus-response relationship for the mechanical responses was compared using two-way ANOVA with repeated measures on one factor followed by post-hoc unpaired t-test. Analysis of the mechanical stimulus–response relationship before and after the chemical stimulus was performed using two-way repeated measures ANOVA followed by post-hoc paired t-tests. Data are presented as mean ± SEM or proportions for each group. Statistical analysis was performed using SPSS 13.0 for Windows (SPSS Inc., Chicago, IL). *p* < 0.05 was considered statistically significant.

## Results

### Isolated DRG Neurons from *ASIC 1a/2/3* Null Mice Lack Transient Acid-evoked Currents

**Figure 1:**
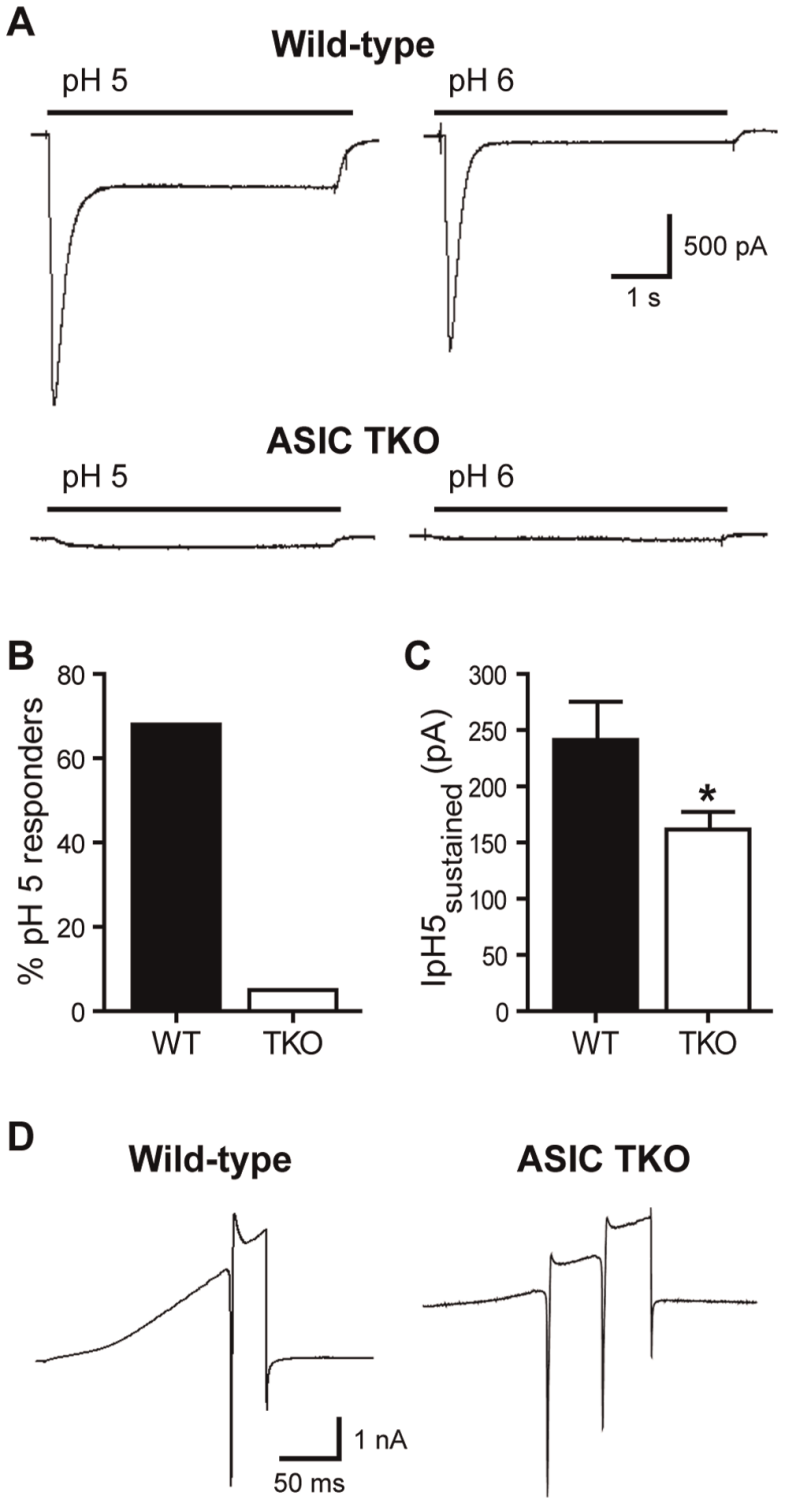
*ASIC*-like currents are absent from *ASIC* triple-knockout (TKO) DRG neurons. ***A***, Representative pH 5- and pH 6-evoked currents from wild-type (WT) and *ASIC* triple-knockout (TKO) DRG neurons. Note that transient acid-evoked currents were never seen from *ASIC* TKO neurons. ***B***, Percentage of DRG neurons from WT and *ASIC* TKO mice that responded to pH 5 with a current amplitude > 250 pA (*n*  =  29 and 32, respectively). ***C***, Mean sustained current amplitudes measured at the end of a 5 s pH application (as in Fig. 1*A*) for above cells. *p < 0.05 *vs*. WT by unpaired t-test. ***D***, Voltage activated currents from WT and *ASIC* TKO mice evoked by ramping from −70 to +20mV in 200 ms.

We first studied acid-evoked responses of isolated DRG neurons from *ASIC* TKO mice and compared them to WT. Figure 1*A* demonstrates typical *ASIC*-like currents recorded from a WT neuron: a rapid drop in pH evoked large rapidly activating and desensitizing (transient) currents that were followed by smaller sustained currents in a majority of cells. Transient currents were recorded in 68% (20 of 29) of WT neurons, and mean amplitude of pH 5-evoked currents was 2310 ± 382 pA. In comparison, acid-evoked transient currents were absent in all *ASIC* TKO neurons studied (*n*  =  32, Fig. 1*B*), and the sustained currents were significantly smaller than those from WTs (Fig. 1*C*). Despite lacking *ASIC*-like currents, DRG neurons from *ASIC* TKO mice displayed normal appearing voltage-activated currents evoked by voltage ramp (Fig. 1*D*).

### Simultaneous Disruption of *ASIC1a*, *2* and *3* Increased the Behavioral Sensitivity to Mechanical Stimuli

**Figure 2:**
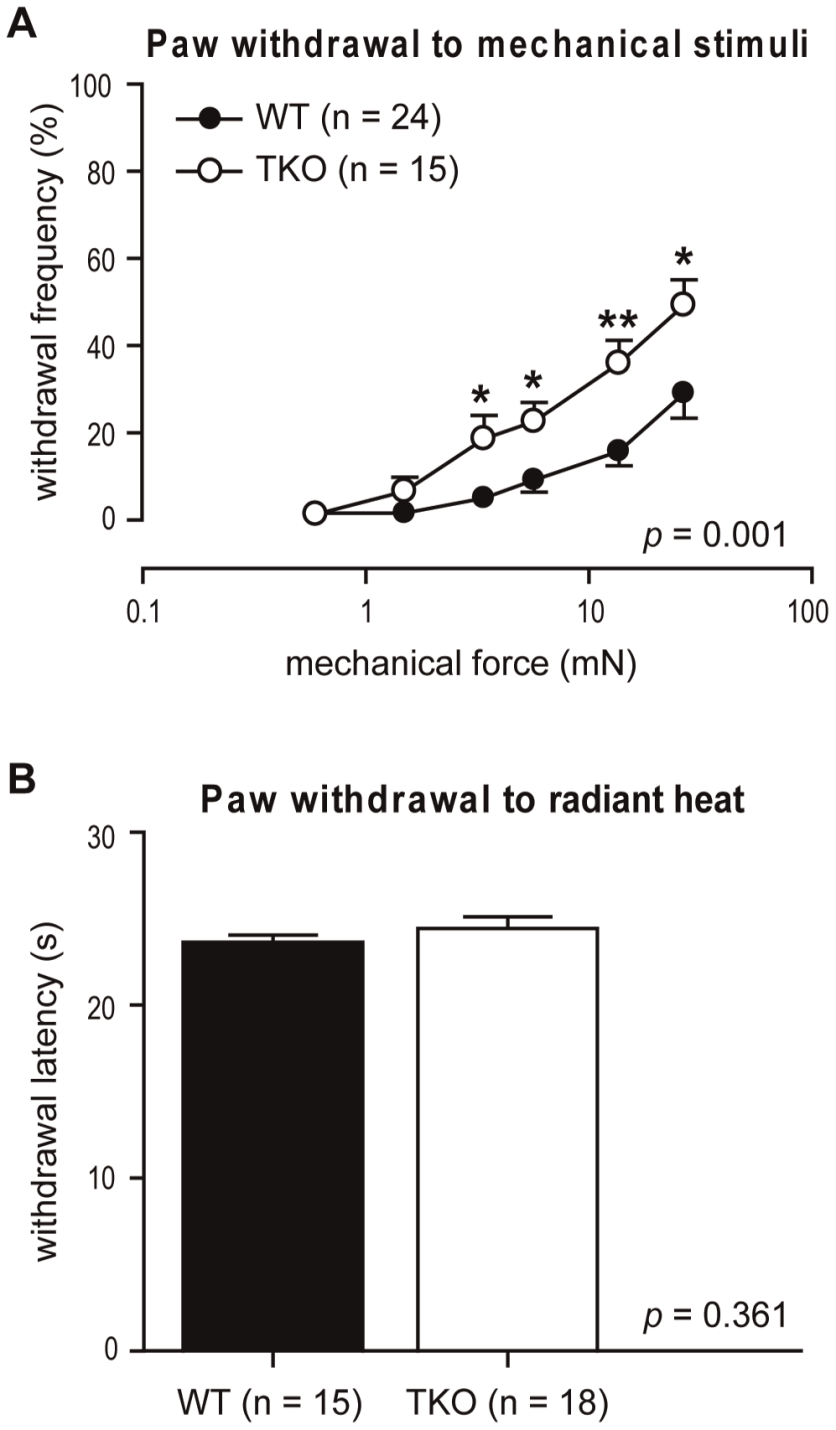
Withdrawal responses to mechanical and heat stimuli in *ASIC* triple-knockout (TKO) and wild-type (WT) mice. ***A***, There was a significant increase in the frequencies of paw withdrawal responses to von Frey filaments in the *ASIC* TKO mice (*n*  =  15, ○) when compared to WT controls (*n*  =  24, •) (*p*  =  0.001 between genotypes by two-way ANOVA with repeated measures on one factor. *p < 0.05; **p < 0.01 *vs*. WT by unpaired t-test). ***B***, Paw withdrawal latency to thermal simulation with radiant heat in *ASIC* TKO (*n*  =  18, ○) and WT mice (*n*  =  15, •). Data are presented as mean ± SEM.

*ASIC* TKO mice showed significantly enhanced mechanosensitivity compared to WT mice, measured as the frequencies of withdrawal responses to the application of von Frey filaments (Fig. 2*A*). When separated by sex, both female (*n*  =  8) and male (*n*  =  7) *ASIC* TKO mice showed increased sensitivity to mechanical stimuli compared to WT mice (12 females and 12 males) (Data not shown). In contrast, the mean withdrawal latency to a slow radiant heat ramp was not statistically different between the two genotypes (Fig. 2*B*).

### Electrophysiological Studies

#### General properties of afferents

**Figure 3:**
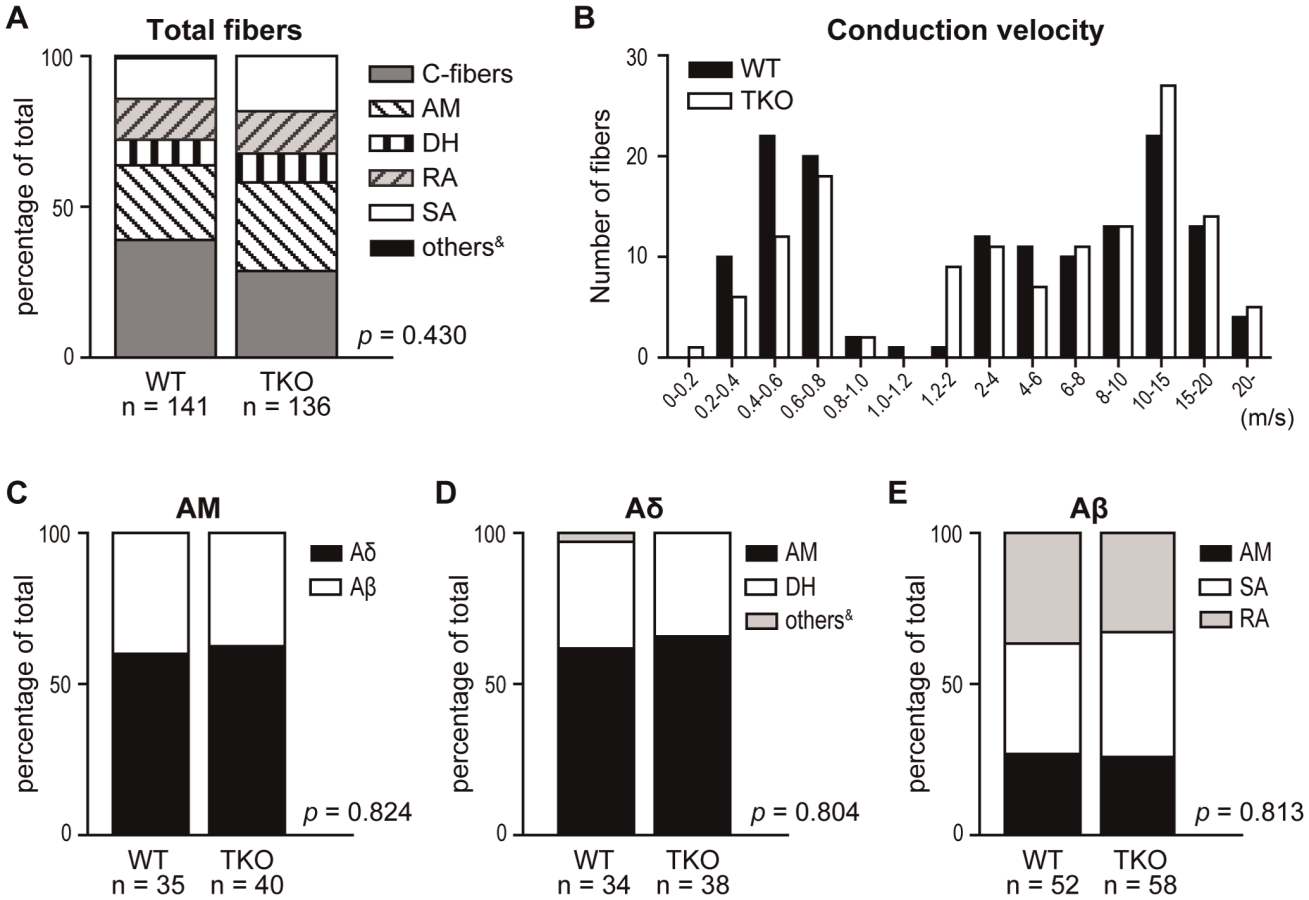
Proportions of the different classes and conduction velocity distribution of mechanosensitive afferents. ***A***, Percentage occurrence of C-fibers, A-mechanonociceptors (AMs), D-hair receptors (DHs), rapidly adapting (RAs) and slowly adapting low-threshold mechanoreceptors (SAs) identified in *ASIC* triple-knockout (TKO) and wild-type (WT) mice. ^&^One slowly adapting Aδ-unit from a WT mouse could not be classified as one of five classes, because it had mechanical threshold of less than 1mN. ***B***, Conduction velocity distribution histogram. ***C***, Percentage of AM that conducted in the Aδ and Aβ conduction velocity range. ***D***, ***E***, Percentage of Aδ- (***D***) and Aβ-fibers (***E***) that were classified as AM, DH, SA or RA.

Extracellular recordings were made from a total of 277 cutaneous primary afferents from 32 mice; 141 fibers were studied from 16 WT control mice (8 female and 8 male) and 136 fibers were studied from 16 *ASIC* TKO (8 female and 8 male) (Table 2 and Fig. 3*A*). Fibers were classified according to their conduction velocity (CV), mechanical threshold and adaptation responses. The proportion of fiber classes was not statistically different between the two genotypes (Fig. 3*A, C–E* and Table 2). The percentage of A-mechanonociceptors (AMs) that conducted in the Aβ range in this study was about 40% in both genotypes, in agreement with data previously reported in rats [22] and guinea pigs [23]. A CV distribution histogram is shown in Figure 3*B*, and the mean CVs of the different fiber classes are shown in Table 2. The mean CV of Aδ-AMs from *ASIC* TKO mice was significantly slower compared to that of Aδ-AMs from WT control (Table 2). There was no difference in the CV of other classes between the two genotypes.

**Table 2:** Proportions and the average conduction velocities (CVs) of the different classes of cutaneous afferents.

C-fibers	WT (*n* = 55)	TKO (*n* = 39)	*p* value
CV (m/s)	0.58 ± 0.17	0.58 ± 0.19	NS
Aδ-fibers	WT (*n* = 34)	TKO (*n* = 38)	*p* value
AM			
%	61.8	65.8	NS
CV (m/s)	4.6 ± 1.6	3.5 ± 1.9*	< 0.05
DH			
%	35.3	34.2	NS
CV (m/s)	5.1 ± 1.6	5.19 ± 1.8	NS
Aβ-fibers	WT (*n* = 52 )	TKO (*n* = 59)	*p* value
AM			
%	27.0	25.4	NS
CV (m/s)	12.8 ± 3.6	12.2 ± 2.8	NS
RA			
%	36.5	32.2	NS
CV (m/s)	15.1 ± 4.7	14.7 ± 5.9	NS
SA			
%	36.5	42.4	NS
CV (m/s)	12.3 ± 3.9	14.7 ± 5.2	NS

Values for CV are means ± SEM. *p < 0.05 *vs*. WT by unpaired t-test.

Abbreviation: AM, A-mechanonociceptor; DH, D-hair receptor; RA, Rapidly adapting low-threshold mechanoreceptor; SA, Slowly adapting low-threshold mechanoreceptor; WT, wild-type C57BL/6J control mice; TKO, *ASIC* triple-knockout mice; NS, not significant.

#### Simultaneous disruption of ASIC1a, -2 and -3 increased the mechanosensitivity of AMs

**Figure 4:**
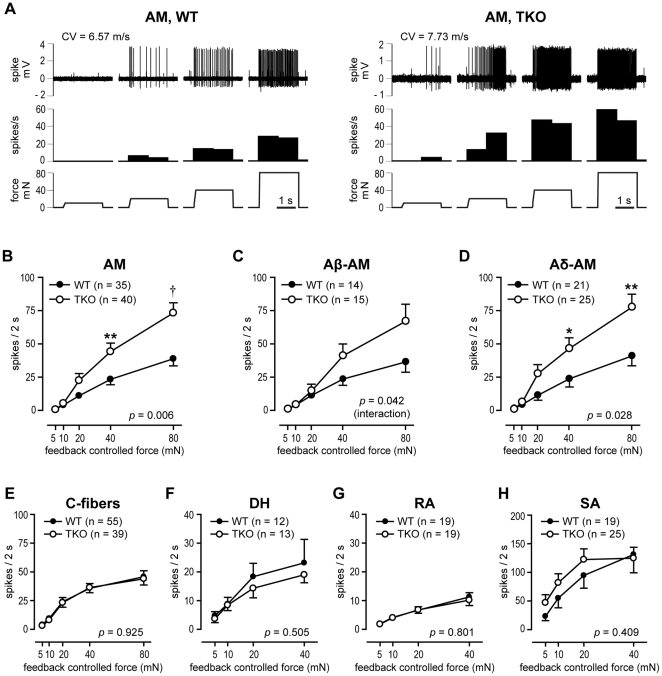
Mechanosensitivity of cutaneous primary afferents in *ASIC* triple-knockout (TKO) and wild-type (WT) mice. ***A***, Sample recording traces showing responses of A-mechanonociceptors (AM) from WT and *ASIC* TKO mice to mechanical stimuli. The *upper, middle and lower panels* show the digitized oscilloscope tracing, the spike density histograms (bin width  =  1 s), and the force stimuli applied, respectively. CV  =  conduction velocity. ***B***, Stimulus-response function of AM from *ASIC* TKO (*n*  =  40, ○) *vs*. WT (*n*  =  35, •), showing enhanced mechanosensitivity in *ASIC* TKO mice (*p*  =  0.006 between genotypes by two-way ANOVA with repeated measures on one factor. **p < 0.01; †*p* < 0.001 *vs*. WT by unpaired t-test). ***C***, ***D***, When AM fibers were subclassified based on CV, not Aβ-AM (***C***) but Aδ-AM (***D***) showed a significant increase in the stimulus-response function in *ASIC* TKO mice (*p*  =  0.028 between genotypes by two-way ANOVA with repeated measures on one factor. *p < 0.05; **p < 0.01 *vs*. WT by unpaired t-test). ***E-H***, Stimulus-response function of C-fibers (***E***), D-hair receptors (DHs) (***F***), rapidly adapting (RAs) (***G***), and slowly adapting low-threshold mechanoreceptors (SAs) (***H***). Data are presented as mean ± SEM.

An ascending series of sustained force stimuli was applied to evaluate the suprathreshold mechanosensitivity. With the exception of the AMs, the stimulus-response functions of all other fiber types were not significantly different between the two genotypes (Fig. 4). The example traces in Figure 4*A* show the responses of AMs to the series of computer-controlled mechanical stimuli. A summary of mechanical responses of AMs is shown in Figure 4*B–D*. When compared to WT control, AMs from *ASIC* TKO showed significantly greater mechanosensitivity (Fig. 4*B*). When AM fibers were subclassified based on their CV in order to further clarify the TKO, Aδ-AMs showed a significant increase in the stimulus-response function in *ASIC* TKO mice compared to WT control (Fig. 4*C, D*). The Aβ-AM fibers also had a significant interaction between force and genotype, and post-hoc tests were not significant at each force. Although the response of Aβ-AM fibers at each force was not statistically different between the genotypes, this may be because these fibers are rare, approximately 10% of all fibers recorded. Another explanation is that disruptions of the *ASIC* genes might have resulted in a greater alteration in the electrophysiological phenotype of Aδ-AMs than Aβ-AMs.

When separated by sex, stimulus-response function showed enhanced mechanosensitivity of AMs from *ASIC* TKOs compared to those from WTs in both female and male mice. The stimulus-responses functions of C-, DH, RA and SA fibers in the *ASIC* TKOs did not differ significantly from their corresponding WT controls in either female or male mice (data not shown).

**Figure 5:**
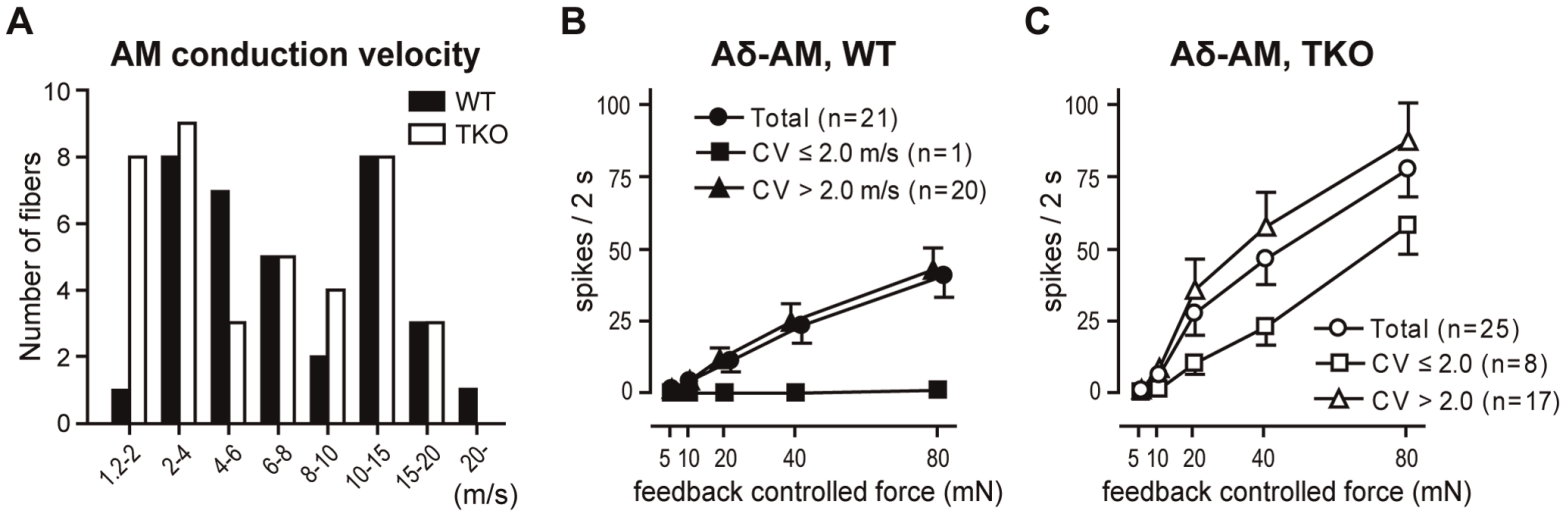
Mechanosensitivity of cutaneous Aδ-mechanonociceptors (Aδ-AM ) in *ASIC* triple-knockout (TKO) and wild-type (WT) mice. Aδ-AM fibers were further subdivided into two groups based on conduction velocity (CV) range (CV ≤ 2.0 *vs*. CV > 2.0). ***A***, Conduction velocity distribution histogram of AM fibers. ***B***, Mechanical stimulus-response function of Aδ-AM from WT mice. Only one fiber conducted slower than 2 m/s (▪). ***C***, Mechanical stimulus-response function of Aδ-AM from *ASIC* TKO mice. Fibers that conducted faster than 2 m/s (*n*  =  17, ▵) showed a tendency toward enhanced mechanosensitivity, compared to those that conducted slower than 2 m/s (*n*  =  8, □) (*p*  =  0.090 between CV groups by two-way ANOVA with repeated measures on one factor). Data are presented as mean ± SEM.

The mean CV of Aδ-AM fibers from *ASIC* TKOs was reduced compared to WTs (Table 2). A conduction velocity histogram revealed an increase in the number of Aδ-AM fibers that conducted relatively slowly (1.2 ≤ CV ≤ 2.0 m/s) in *ASIC* TKOs (Fig. 3*B*). While only 1 of 21 Aδ-AMs from WTs conducted at velocities of 1.2 to 2.0 m/s, 8 of 25 from *ASIC* TKOs conducted at this velocity range (Fig. 5*A*). To test whether these slower Aδ-AM fibers in *ASIC* TKOs contributed to an enhanced mechanosensitivity, we subdivided Aδ-AMs into two CV groups (≤ 2.0 *vs*. > 2.0), and compared mechanical stimulus-response functions (Fig. 5*B, C*). As shown in Figure 5*C*, faster fibers (CV > 2 m/s), rather than slower fibers (CV ≤ 2 m/s), contributed to the enhanced mechanosensitivity of Aδ-AMs from *ASIC* TKOs. Therefore, it is unlikely that decreased CV of Aδ-AMs in *ASIC* TKOs is directly related to the enhanced mechanosensitivity.

**Figure 6:**
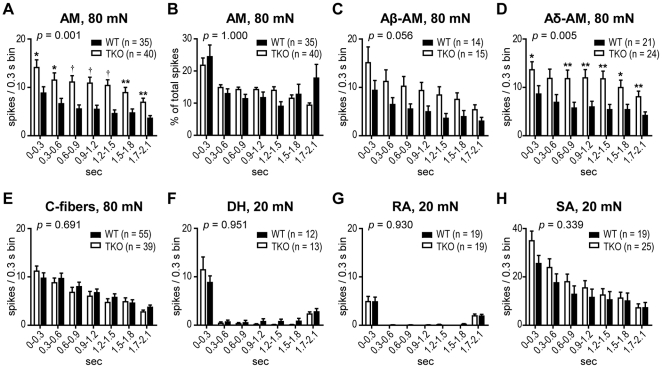
Adaptation properties of cutaneous primary afferents in *ASIC* triple-knockout (TKO) and wild-type (WT) mice. ***A***, AM from *ASIC* TKO mice showed increased firing initially and throughout the duration of sustained, 80-mN stimulus (*P*  =  0.001 between genotypes by two-way ANOVA with repeated measures on one factor. *P < 0.05; **P < 0.01; †*P* < 0.001 *vs*. WT by unpaired t-test). ***B***, When the action potentials in each 0.3-s bin were normalized to total spikes generated during the entire duration of stimulus, there was no significant difference between the two genotypes. ***C***, ***D***, When AM fibers were subclassified based on conduction velocity, not Aβ-AM (***C***) but Aδ-AM (***D***) from *ASIC* TKO mice showed a significantly increased firing initially and throughout the duration of the stimulus (*p*  =  0.005 between genotypes by two-way ANOVA with repeated measures on one factor. *p < 0.05; **p < 0.01 *vs*. WT by unpaired t-test). ***E-H***, Average action potentials in 0.3-s bins during a sustained force stimulus in C-fibers (***E***), D-hair receptors (DHs) (***F***), rapidly adapting (RAs) (***G***), and slowly adapting low-threshold mechanoreceptors (SAs) (***H***). Data are presented as mean ± SEM.

Adaptation properties of each fiber class were assessed by comparing the average action potentials in 0.3-s bins during a sustained, mechanical force stimulus. Binned data from the 80 mN stimulus were used for mechanonociceptors (C-fibers and AMs), and 20 mN force was used for low-threshold mechanoreceptors (DH, RA and SA fibers). AMs from *ASIC* TKO mice showed significantly increased firing initially and during the entire duration of the 80 mN stimulus, compared to WT control (Fig. 6*A*). When the action potentials in each bin were normalized to total action potentials generated during the entire duration of stimulus, there was no significant difference between the two genotypes (Fig. 6*B*). When AM fibers were subclassified based on their CV, Aδ-AMs but not Aβ-AMs from *ASIC* TKO mice showed significantly increased firing initially and throughout the duration of the stimulus, compared to WT controls (Fig. 6*C, D*). C-, DH, RA and SA fibers did not show any statistically significant differences in the adaptation properties between the two genotypes (Fig. 6*E–H*).

#### The effects of acid on mechanical responses of cutaneous primary afferents

**Figure 7:**
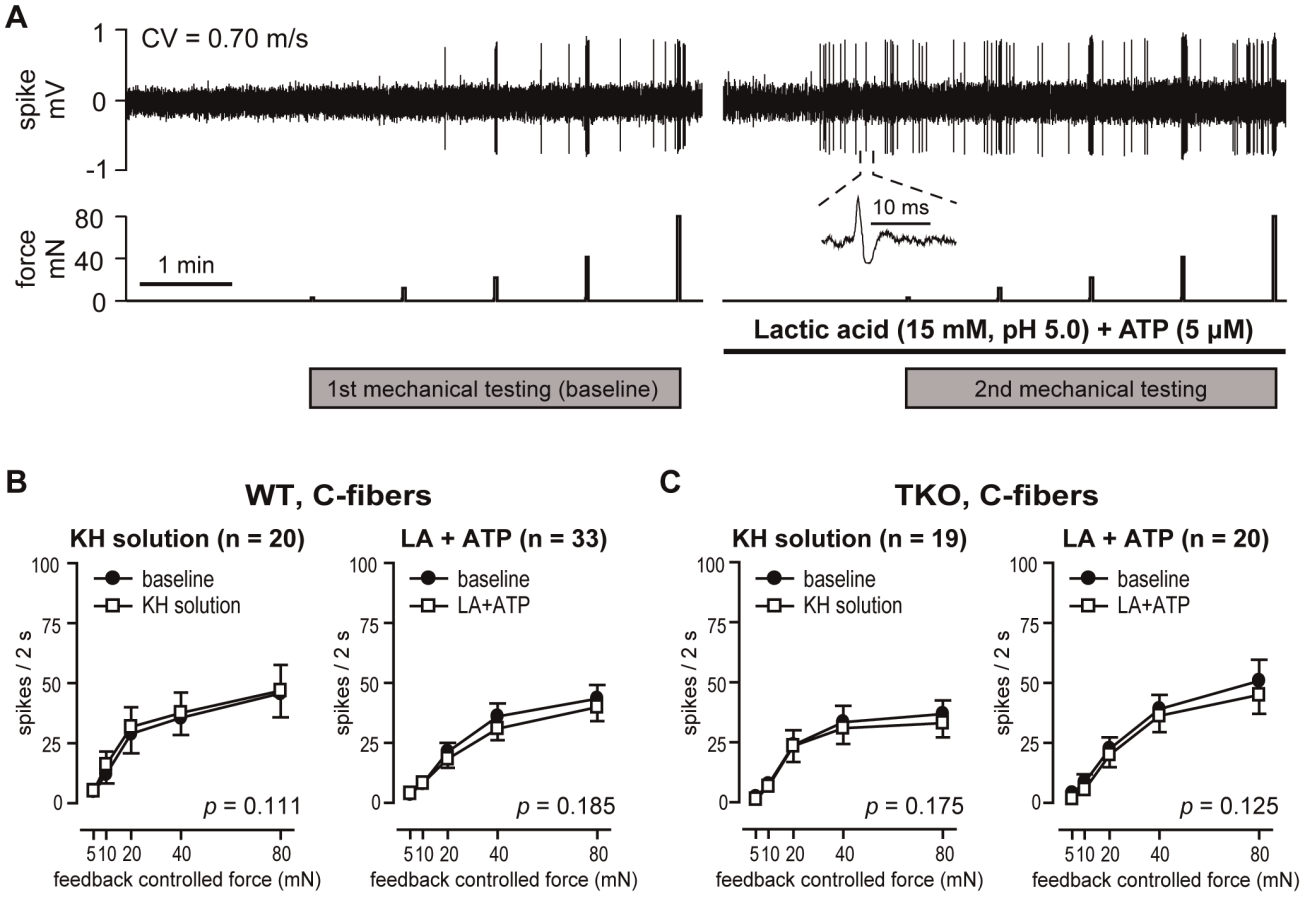
Chemosensitivity and the effects of lactic acid plus ATP on mechanical responses of C-fibers. ***A***, Sample recording from a single C-fiber from a wild-type (WT) mouse. After first series of force stimuli was applied to the receptive field, the modified K-H solution inside the ring was replaced with either lactic acid/ATP or control K-H solution. Then, after 2-min application of chemical solution, the same series of mechanical stimuli was applied in the presence of applied solution. The *upper and lower panels* show the digitized oscilloscope tracing and the force stimuli applied, respectively. *Inset* displays the action potentials of this unit. CV  =  conduction velocity. ***B***, ***C***, Mechanical stimulus-response function of C-fibers from WT (***B***) and *ASIC* triple-knockout (TKO) mice (***C***) before and during exposure to pH 5.0 lactic acid plus ATP or control K-H solution (modified Krebs-Henseleit solution equilibrated with room air). Data are presented as means ± SEM. LA  =  lactic acid; K-H  =  Krebs-Henseleit.

**Figure 8:**
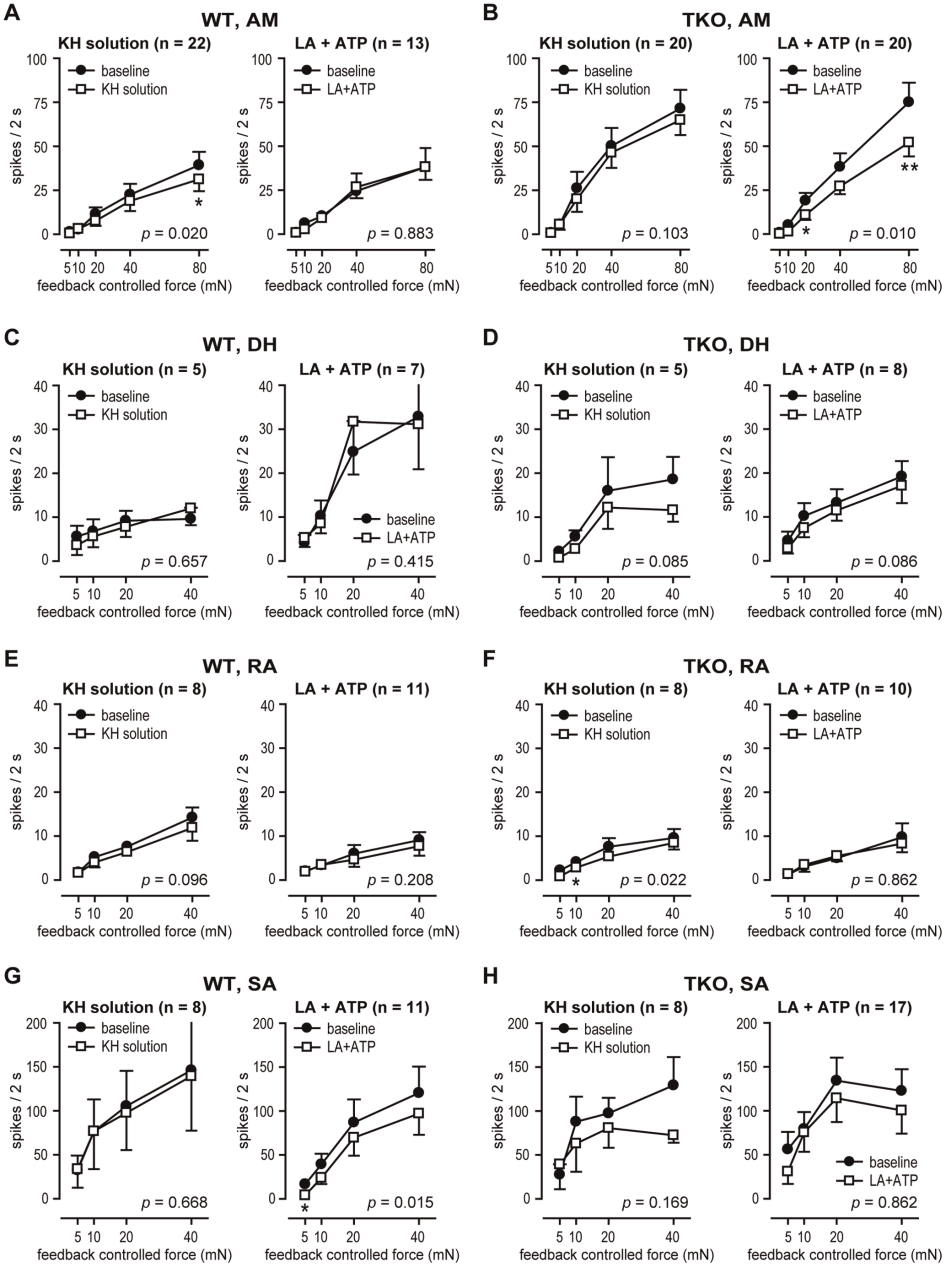
Effects of lactic acid plus ATP on mechanical responses of cutaneous afferents. ***A-H***, Mechanical stimulus response function of A-mechanonociceptors (AM), D-hair receptors (DHs), rapidly adapting (RAs) and slowly adapting low-threshold mechanoreceptors (SAs) before and during exposure to pH 5.0 lactic acid plus ATP or control K-H solution. *p < 0.05; **p < 0.01 *vs*. baseline by paired t-test. Data are presented as means ± SEM. WT  =  wild-type. TKO  =  triple-knockout. LA  =  lactic acid; K-H  =  Krebs-Henseleit.

The sample recording in Figure 7*A* shows the experimental protocol used to test chemosensitivity and the effect of pH 5.0 lactic acid/ATP on mechanical responses of afferents. A C-fiber from a WT mouse in this example was a chemosensitive unit that was activated during application of pH 5.0 lactic acid/ATP. Four of 33 C-fibers (12.1%) from WT control mice and none of 20 C-fibers (0%) from *ASIC* TKO mice were responsive to the chemical stimulus. No AM, DH, RA or SA fiber in either genotype was chemosensitive. C-fiber responses to mechanical stimuli were not sensitized or desensitized by pH 5.0 lactic acid/ATP or the control K-H solution in either genotype (Fig. 7*B,C*). Mechanical stimulus response function curves of AM (Fig. 8*A, B*), DH (Fig. 8*C, D*), RA (Fig. 8*E*, F) and SA (Fig. 8*G, H*) fibers before and during exposure to pH 5.0 lactic acid/ATP or the control K-H solution are shown in Figure 8. In some fiber groups from both genotypes, a slightly decreased mechanosensitivity was noted during exposure to the test or control solutions, suggesting some degree of fatigue (Fig. 8*A, B, F, G*). It is unlikely that this change is related to alteration in skin compliance because C-fibers maintained their mechanical stimulus-response properties during the second stimulus.

## Discussion

### *ASIC* Gene Disruption and Mechanosensitivity

Combined knockout of *ASIC1a*, -*2* and -*3* increased the behavioral sensitivity to mechanical stimuli and the mechanosensitivity of AMs compared to WTs. The responses of all other fiber types were not different between the two genotypes.

Earlier work localized *ASIC* subunits in specialized cutaneous mechanosensory structures [8]–[10], and sequence comparisons showed similarity between *ASIC* subunits and the mechanosensory Mec-4 and Mec-10 subunits of *C. elegans* [4], [5]. Therefore, we hypothesized that disrupting all three *ASIC* genes would markedly impair mechanosensation. We found just the opposite; enhanced mechanosensitivity in *ASIC* TKO mice. One possible explanation for this intriguing discovery could be that *ASIC* channels are mechanosensors, but that they somehow reduce or inhibit mechanosensitivity. An inhibitory mechanism is consistent with a previous study demonstrating enhanced mechanosensitivity in visceral afferents from *ASIC1* null mice using single-fiber nerve recordings [11]. Yet other studies have shown that mechanical displacement of *ASIC*-expressing neurons generated greater depolarization compared to *ASIC*-nonexpressing neurons [24]. *ASIC* activation depolarizes the cell membrane [25], [26], consistent with their Na^+^ and, to a lesser extent, Ca^2+^ permeability [27], [28]. That, plus their peripheral localization would argue that if they were mechanoreceptors, they would activate rather than inhibit mechanosensory neurons.

We speculate that in the sensory extensions of DRG neurons, *ASIC*s interact with and dampen the function of either mechanosensory channels or molecules required to generate maximal neuronal activity following a mechanical stimulus. Thus, disruption of *ASIC* genes might enhance mechanosensitivity. Previous studies have shown that *ASIC* subunits associate with a variety of scaffolding proteins, including PICK1 [29], PSD-95 [30], [31], Lin-7b [30], α-actinin [32], CIPP [33], NHERF-1 [34], MAGI-1b, PIST [30], and stomatin [35]. In addition, *ASIC*s physically and/or functionally interact with other membrane ion channels that have been associated with mechanosensation, including BK channels [36], P2X channels [37], and TRP channels [17], [38], [39].

Although our conclusion that *ASIC*s contribute to mechanosensation is consistent with some previous data, the phenotypes of the *ASIC* TKO mice could not have been predicted from studies using *ASIC* single-knockout mice. For example, *ASIC* TKOs showed increased paw withdrawal frequencies to a range of von Frey filaments, suggesting enhanced behavioral mechanosensitivity. In contrast, *ASIC* single-knockout mice had no alteration in paw withdrawal responses to these stimuli [10], [11], [14]. Our data are consistent with a previous behavioral study of transgenic mice expressing a dominant-negative *ASIC3* subunit that was predicted to inactivate all *ASIC*-like transient currents [40]; transgenic mice exhibited a decreased paw withdrawal threshold to von Frey filaments. On the other hand, paw withdrawal frequencies to von Frey filaments were not different between these transgenic mice and WTs under non-pathological conditions. Our data demonstrated greater paw withdrawal frequencies in *ASIC* TKOs compared to WTs, suggesting further enhanced mechanosensitivity in *ASIC* TKOs. In addition, the enhanced mechanosensitivity of *ASIC* TKO AM fibers could not have been predicted from studies using *ASIC* single-knockout mice. As stated in the Introduction section, previous studies have demonstrated reduced mechanosensitivity of RAs and SAs in *ASIC2* nulls [9], enhanced mechanosensitivity of RAs and reduced mechanosensitivity of Aδ-AMs in *ASIC3* nulls [10], and no difference in the mechanosensitivity of cutaneous afferents in *ASIC1* nulls [11]. Thus, our results suggest some functional redundancy of *ASIC* subunits in conferring a normal mechanosensory phenotype. *ASIC* channels are composed of three *ASIC* subunits, and in DRG neurons, previous data suggest that *ASIC* channels are predominantly heteromultimers [7]. Our data together with earlier studies suggest that any two of the subunits may be sufficient to confer normal mechanosensation.

Other potential mechanisms that could be considered for the enhanced mechanosensitivity in *ASIC* TKOs are as follows. First, *ASIC1a* and *ASIC2* subunits are expressed in the central nervous system (CNS) [18], [41], [42]. It is possible that *ASIC* channels in brain might play an inhibitory role in the response to mechanosensory stimuli, and hence their loss might enhance the behavioral response to peripheral stimulation. While that could be the case, our finding that loss of *ASIC* function increased mechanical responsiveness to a specific fiber population in a preparation lacking connections to the CNS, and even to cell bodies in the DRG, argues that loss of *ASIC* subunits had a more direct effect on the mechanosensory process. If *ASIC*s in the CNS were inhibiting behavioral responsiveness, heat latencies should have been affected. Second, it remains possible that there could be functional redundancies between *ASIC*s and other candidate mechanosensory channels including transient receptor potential (TRP) channels, two-pore-domain K^+^ (K_2P_) channels [43] and piezo proteins [44]. Perhaps the loss of *ASIC* leads to compensatory changes in other mechanosensory channels. However, if that were the case, it would require “overcompensation” by other channels to produce the enhanced mechanosensitivity in *ASIC* TKO mice. Third, the increased mechanosensitivity in AM fibers is not likely caused by differences in skin compliance or thickness because the mechanosensitivity of other fibers was not affected.

### Behavioral Responses and Afferent Fiber Activity

Our data suggest that increased AM fiber mechanosensitivity is responsible for the increased paw withdrawal frequencies to punctate mechanical stimuli. Although it is not clear whether the paw withdrawal response to von Frey filaments is related to a painful sensation [45], it has been suggested that this test in rodents reflects the response of nociceptive primary afferents [10], [46]. In a human study, selective conduction blockade of A-fibers by non-ischemic nerve compression significantly reduced the magnitude of pain elicited by punctate mechanical probes in normal skin [47]. Similarly, in neonatal rats treated with 50 mg/kg of capsaicin, 95% of C-fibers were destroyed [48], [49], and the paw withdrawal responses to punctate mechanical stimuli of these rats were not different from normal rats, implying a minimal role of C-fibers in this assay [50]. Thus, the behavioral and electrophysiological responses in *ASIC* TKOs may represent an increased response to a noxious mechanical stimulus.

Consistent with the increased mechanosensitivity of the AM fibers, *ASIC2* and *ASIC3* immunoreactivities were detected in the intraepidermal free nerve endings [8], [10]. On the other hand, while previous studies have shown *ASIC2* and *ASIC3* immunoreactivities in specialized cutaneous mechanosensory structures [8]–[10], mechanosensitivity of low-threshold mechanoreceptors in *ASIC* TKOs did not differ significantly from their corresponding WT controls.

Considering that different *ASIC* subunits are expressed in various subsets of DRG neurons [6], it is unclear why the altered mechanosensitivity was only observed in AMs but not other fibers in *ASIC* TKOs. First, it could be that disrupting *ASIC1a*, -*2* and -*3* genes might affect some other channel/process that is unique to AM neurons. Second, we cannot absolutely exclude some contribution of *ASIC1b*. However, although *ASIC1b* mRNA was detected in mice with a disruption of *ASIC1a*, no evidence of either *ASIC1a* or *ASIC1b* protein was found in these animals [11]. In addition, no transient acid-evoked currents were found in *ASIC* TKO DRG neurons, suggesting that presence of *ASIC1b* is very unlikely. Third, while the feedback-controlled constant-force mechanical stimulus used in the present study is optimal for evaluating nociceptors [21], [51], it might have limited sensitivity for detecting differences in RA or SA responses, mainly velocity- or displacement-detectors [52]. Because fiber classifications were finalized after the complete array of mechanical stimuli, we could only apply one modality and we selected force. This should also be considered when comparing mechanosensitivity data among studies, since displacement-controlled stimuli were used in the previous *ASIC* single-null mice studies [9]–[11].

### *ASIC*s and Chemosensitivity

Chemosensitivity of primary afferents was assessed using pH 5.0 lactic acid/ATP. Lactate and ATP were added in an attempt to optimize the *ASIC*-medicated responses of primary afferents to low pH [17], [37], [53], [54]. We found very little activation of sensory fibers by lactic acid/ATP and no potentiation of mechanical sensitization in acidic conditions. Previously, in a study using the rat skin-nerve preparation, Steen et al. reported low-pH-induced sensitization of nociceptors to mechanical stimulation [55]. These seemingly opposite findings are likely attributed to the differences in the experimental protocols, including the frequency and duration of low-pH exposure. Also, the differences in species and the composition of the acid solution might have contributed to the discrepancy [55].

### Conclusion and Future Studies

Combined knockout of *ASIC1a*, -*2* and -*3* increased the behavioral sensitivity to mechanical stimuli and the mechanosensitivity of AMs. Our data indicate that *ASIC*s are involved in cutaneous mechanosensation. Future studies seeking to understand why *ASIC* TKOs have enhanced mechanosensitivity, such as identifying transcripts that are up- or down- regulated in AMs from TKOs, may help identify other components of the mechanosensory complex and evaluate possible compensatory effects. It would be also interesting for future studies to investigate mechanically activated current in dissociate DRG neurons to characterize other mechanosensory phenotypes of *ASIC TKO*s. We have shown slower mean conduction velocity (CV) of Aδ-AMs from *ASIC* TKOs. Previous studies have demonstrated the expression of *ASIC2* or other channels from DEG/ENaC superfamily by human or rat Schwann cells [56], [57]. Therefore, while the exact underlying mechanism remains unclear, warranting further investigation, this might suggest a possible role of *ASIC*s in Schwann cell function and axonal conduction in sub-populations of afferents.
